# Designer synthetic media for studying microbial-catalyzed biofuel production

**DOI:** 10.1186/s13068-014-0179-6

**Published:** 2015-01-22

**Authors:** Xiaoyu Tang, Leonardo da Costa Sousa, Mingjie Jin, Shishir PS Chundawat, Charles Kevin Chambliss, Ming W Lau, Zeyi Xiao, Bruce E Dale, Venkatesh Balan

**Affiliations:** Biogas Institute of Ministry of Agriculture, Section 4-13 Remin South Road, Chengdu, 610041 P. R. China; DOE Great Lakes Bioenergy Research Center, Biomass Conversion Research Lab (BCRL), Chemical Engineering and Materials Science, Michigan State University, 3815 Technology Boulevard, Suite 1045, Lansing, 48910 USA; Department of Chemical & Biochemical Engineering, Rutgers, The State University of New Jersey, 98 Brett Road, Room C-150A, Piscataway, NJ 08854 USA; Department of Chemistry and Biochemistry, Baylor University, Waco, TX 76798 USA; School of Chemical Engineering, Sichuan University, No. 24 South Section 1, Yihuan Road, Chengdu, 610065 P. R. China

**Keywords:** Synthetic hydrolysate, Lignocellulose, AFEX, Yeast fermentation inhibition, Amides inhibition, Carboxylic acids inhibition, Pretreatment decomposition products, Hydrolysate composition

## Abstract

**Background:**

The fermentation inhibition of yeast or bacteria by lignocellulose-derived degradation products, during hexose/pentose co-fermentation, is a major bottleneck for cost-effective lignocellulosic biorefineries. To engineer microbial strains for improved performance, it is critical to understand the mechanisms of inhibition that affect fermentative organisms in the presence of major components of a lignocellulosic hydrolysate. The development of a synthetic lignocellulosic hydrolysate (SH) media with a composition similar to the actual biomass hydrolysate will be an important advancement to facilitate these studies. In this work, we characterized the nutrients and plant-derived decomposition products present in AFEX™ pretreated corn stover hydrolysate (ACH). The SH was formulated based on the ACH composition and was further used to evaluate the inhibitory effects of various families of decomposition products during *Saccharomyces cerevisiae* 424A (LNH-ST) fermentation.

**Results:**

The ACH contained high levels of nitrogenous compounds, notably amides, pyrazines, and imidazoles. In contrast, a relatively low content of furans and aromatic and aliphatic acids were found in the ACH. Though most of the families of decomposition products were inhibitory to xylose fermentation, due to their abundance, the nitrogenous compounds showed the most inhibition. From these compounds, amides (products of the ammonolysis reaction) contributed the most to the reduction of the fermentation performance. However, this result is associated to a concentration effect, as the corresponding carboxylic acids (products of hydrolysis) promoted greater inhibition when present at the same molar concentration as the amides.

Due to its complexity, the formulated SH did not perfectly match the fermentation profile of the actual hydrolysate, especially the growth curve. However, the SH formulation was effective for studying the inhibitory effect of various compounds on yeast fermentation.

**Conclusions:**

The formulation of SHs is an important advancement for future multi-omics studies and for better understanding the mechanisms of fermentation inhibition in lignocellulosic hydrolysates. The SH formulated in this work was instrumental for defining the most important inhibitors in the ACH. Major AFEX decomposition products are less inhibitory to yeast fermentation than the products of dilute acid or steam explosion pretreatments; thus, ACH is readily fermentable by yeast without any detoxification.

**Electronic supplementary material:**

The online version of this article (doi:10.1186/s13068-014-0179-6) contains supplementary material, which is available to authorized users.

## Background

Increasing fossil fuel utilization by industrialized economies has been a subject of intense debate within the scientific and political communities worldwide. Increasing energy demand, depleting petroleum reserves, the negative environmental repercussions due to increased greenhouse gas (GHG) emissions, and the control of fossil fuel production by a limited number of nations are among the main reasons why there is an ongoing effort to restructure our energy sector towards greater sustainability via utilization of renewable sources of energy [1].

Lignocellulosic biofuels are projected to play a substantial role in the replacement of current-generation fossil-derived liquid fuels such as gasoline and diesel [2,3]. In second generation biorefineries, ethanol production from lignocellulosic substrates involves enzymatic digestion of cellulose and hemicellulose sugar polymers into fermentable sugars, which can be converted to ethanol during microbial fermentation. However, the plant cell wall structure has naturally evolved to be highly recalcitrant to enzymatic deconstruction by fungi and bacteria. In order to improve enzyme accessibility to the polysaccharides embedded in plant cell walls, some form of pretreatment is necessary to reduce biomass recalcitrance to enzymatic hydrolysis.

Among the pretreatment technologies available today, thermochemical pretreatments are considered to be the most promising [4]. Most of these pretreatment processes use either acids (such as sulfuric acid, phosphoric acid, and maleic acid) or bases (such as ammonium hydroxide, sodium hydroxide, and potassium hydroxide) to pretreat plant cell walls, often resulting in the formation of cell wall-derived decomposition products that can inhibit both enzymes and microbes [5-7].

Ammonia Fiber Expansion (AFEX™)^a^ is a well-established pretreatment technology that utilizes concentrated ammonia at relatively low temperatures (60 to 140°C) and short residence times (5 to 45 min) to pretreat biomass [4]. AFEX has proven to be particularly effective on monocot-based grasses (for example, corn stover), improving cellulose hydrolysis rates by up to fivefold and generating highly fermentable hydrolysates [8]. Moreover, AFEX produces much lower concentrations of sugar-derived decomposition products compared to acidic pretreatments, while preserving the native nutrient content for more efficient fermentation [9-11]. Therefore, AFEX-based biomass hydrolysates do not require detoxification, exogenous nutrient supplementation, and extensive water washing of the pretreated substrate for efficient glucose fermentation by yeast or bacteria [10,12]. However, the efficiency of xylose consumption during co-fermentation of AFEX pretreated biomass hydrolysates (enriched in both pentoses and hexoses) still requires improvement. Some of the issues faced during mixed hexose/pentose fermentation are the low xylose consumption rate and the lower ability of yeast and bacteria to co-ferment hexose/pentose mixtures [10,12]. Our recent work in *Escherichia coli* KO11 and *Saccharomyces cerevisiae* 424A (LNH-ST) demonstrated that the xylose consumption rate is related to the presence of pretreatment-derived biomass decomposition products, ethanol, and other fermentation metabolites [13]. In the case of *E. coli* KO11, the ability to consume xylose from AFEX hydrolysate was severely affected by the presence of pretreatment-derived biomass degradation products in combination with high concentrations of ethanol. On the other hand, a 22% reduction of cell growth and 13% reduction of specific xylose consumption rate was observed for *S. cerevisiae* 424A (LNH-ST) due to the presence of AFEX decomposition products in the hydrolysate. However, very little is known about the nature of pretreatment-based biomass decomposition products that inhibit xylose consumption, their mechanism of action, and their overall effect on the metabolism of sugars by yeast and bacteria. Answering these questions is an important step toward developing new microbial strains with improved performance on lignocellulosic hydrolysates, and hence increasing the economic competitiveness of liquid biofuels as a viable substitute to conventional gasoline and diesel.

One approach for gaining a deeper understanding of the interactions between inhibitory components present in biomass hydrolysates and microorganisms, including inhibition synergies, levels of inhibition, and metabolic effects, involves using a synthetic medium that mimics the composition of authentic lignocellulosic hydrolysates, that is, a synthetic hydrolysate (SH). The importance of such SHs for these studies is supported by the work published by Lau and Dale (2009) [10], who observed that the inhibition of xylose fermentation is closely dependent on the nutrient availability in the culture medium. The formulation of an SH will enable the inclusion of precisely defined positive and negative controls in experimental designs, which represent a current limitation of directly using complex lignocellulosic hydrolysates. Also, using an SH will allow the manipulation of relative concentrations and ratios between the different components of the hydrolysate, according to the objective of each study. Furthermore, the SH will facilitate the integration of isotope-labeled components in the medium (for example, ^13^C-labeled xylose or glucose) to conduct metabolomics-based experiments, aiming to trace potential deviations in the metabolic flux during xylose consumption in the presence and absence of compounds of interest.

In this work, we have attempted to establish a platform for conducting the above-mentioned studies, by characterizing a highly complex lignocellulosic hydrolysate derived from AFEX pretreated corn stover (AFEX-CS) and formulating a well-defined SH using both commercially available and custom-synthesized reagents/chemicals. This SH platform was also implemented here to screen the effect of different classes of AFEX pretreatment-based biomass decomposition products on xylose fermentation using a recombinant *S. cerevisiae* 424A (LNH-ST) strain.

## Methods

### Biomass

Corn stover (CS) was harvested at Field 570-C Arlington Research Station, University of Wisconsin, in the year 2008. Pioneer 36H56 (triple stack - corn borer/rootworm/Roundup Ready) seeds were used for planting. The CS sample containing leaves, stem, and cobs was dried to < 8% moisture (dry weight basis) using a 60°C oven and milled to 4-mesh size and stored in sealed polythene bags at room temperature until further use. The composition of the untreated corn stover (UT-CS) was 35.7% glucan, 21.2% xylan, 2.6% arabinan, 17.4% lignin, 5.9% ash, and 2.4% acetyl content. AFEX pretreatment was carried out using the procedure reported by Balan *et al.* [14]. The pretreatment condition in this study was 1:1 ammonia to biomass ratio (dry weight), 60% moisture loading, and 140°C for 15 min total residence time. After pretreatment, the residual ammonia was allowed to evaporate in the hood overnight, before being bagged and stored at 4°C prior to further usage. The composition of the pretreated biomass did not change appreciably as a result of the AFEX pretreatment [15].

### Chemicals

#### Feruloyl amide

Feruloyl amide was synthesized via a one-step ammonolysis reaction, using ethyl 4-hydroxy-3-methoxycinnamate (AK Scientific, Inc., Mountain View, CA, USA) as the starting reagent. For the reaction, 3 g of ethyl 4-hydroxy-3-methoxycinnamate was dissolved in 150 mL of 28 to 30% ammonium hydroxide (EMD, Gibbstown, NJ, USA) solution in a high pressure reactor (HEL, Inc., Lawrenceville, NJ, USA). The reaction was carried out at 100°C for 5 h at 300 rpm mixing speed. Under these conditions feruloyl amide was the major product, followed by ferulic acid. The purification and recovery of feruloyl amide were conducted by preparative-scale HPLC using a Waters XBridgeTM Prep C18 column (5.0 μm, 10 mm × 100 mm; Waters Co., Milford, MA, USA). The HPLC system was equipped with a Waters 600 Controller, Waters Delta 600 pump, and a Shimadzu SPD-M10A VP Diode Array Detector and connected to a Waters Fraction Collector (Waters Co., Milford, MA, USA). The solution was diluted to about 5 g/L of feruloyl amide in methanol before injection. The injection volume was 5 mL, and the HPLC flow rate was 0.25 mL/min using the gradient shown in Table 1. The feruloyl amide enriched fractions were freeze dried to a powder and stored in a desiccator. Fractions with purity >95% (as determined by LC-MS and described in Chundawat et al. [15]) were pooled together before utilization in fermentation experiments.

**Table 1:** **HPLC mobile phase and gradient used for isolation of phenolic amides**

**Time**	**0.1%** **formic acid**	**100%** **methanol**
**(min)**	**(% Solvent A)**	**(% Solvent B)**
0	95	5
1.00	95	5
10.00	70	30
18.00	50	50
25.00	50	50
25.01	95	5
30.00	95	5

#### Coumaroyl amide

Coumaroyl amide was also synthesized using the same methodology as for feruloyl amide. However, in this case the ammonolysis reaction was carried out on methyl 4-hydroxycinnamate (Frinton Laboratories, Inc., Vineland, NJ, USA). The purification and recovery of coumaroyl amide were performed using the same methodology as for feruloyl amide. Fractions with purity >95% (as determined by LC-MS and described in Chundawat *et al.* [15]) were pooled together before utilization in fermentation experiments.

#### Xylo-oligomers

The xylo-oligomer mixture was produced by enzymatic hydrolysis of Birchwood xylan (Sigma-Aldrich, St. Louis, MO, USA) using an NS50014 series endoxylanase enzyme (5 mg/mL protein concentration estimated by the Kjeldahl method) provided by Novozymes (Davis, CA, USA). The enzyme composition and substrate specificity for this cocktail have been provided elsewhere [16]. The reaction was carried out at 6% (w/v) solids loading in a 250-mL flask at pH 4.8 (0.05 M phosphate buffer), 150 rpm, and 50°C for 48 h, with an enzyme loading of 2 mL/g xylan. The supernatant was separated from the undissolved solids by centrifugation to further isolate the soluble xylo-oligomers. A Thermo Scientific Hypersep Hypercarb PGC 453 column (Thermo Scientific, Bellefonte, PA, USA) was employed to separate the xylo-oligomers from other soluble products. The column was first conditioned with 30 mL of methanol followed by 30 mL of distilled water. A sample volume of 3.5 mL of hydrolysate was then added to the column. After washing the column with 45 mL of water, 60 mL of methanol was added to elute xylo-oligomers from the sorbent. Methanol was removed using a rotary evaporator (BUCHI, Switzerland) and the xylo-oligomer solution was adjusted to appropriate concentrations in distilled water.

#### Other chemicals

With the exception of the compounds described above, all other chemicals used in the SH were purchased from various commercial vendors: formic acid, 4-hydroxybenzaldehyde, *trans*-aconitic acid, vanillic acid, and vanillin were obtained from Fluka (St. Louis, MO, USA), and all other chemicals were purchased from Sigma-Aldrich (St. Louis, MO, USA). Cellobiose was used as the only gluco-oligomeric component.

### Preparation of AFEX-CS hydrolysate (ACH)

AFEX-CS was enzymatically hydrolyzed with a commercial enzyme mixture as previously described [10]. The enzyme mixture was composed of Spezyme™ CP (79.6 mL/kg CS; protein concentration: 88 mg/mL), Novozyme™ 188 (40.1 mL/kg CS; protein concentration: 150 mg/mL), Multifect Xylanase (11.6 mL/kg CS; protein concentration: 35 mg/mL), and Multifect Pectinase (8.2 mL/kg CS; protein concentration: 90 mg/mL). Spezyme™ CP and Multifect enzyme cocktails were obtained from Genencor Inc., while Novozyme™ 188 was procured from Sigma-Aldrich Co. The glucan loading used for biomass hydrolysis was 6% by weight, which was equivalent to about 19% solids loading. The enzymatic hydrolysis was performed in a 3-L glass autoclavable bioreactor equipped with ez-Control (Applikon Biotechnology B.V., Schiedam, Netherlands) at 50°C and 1,000 rpm for 96 h. A total of 2.5 kg of reaction contents (biomass, water, enzymes, and antibiotics) was loaded into the reactor with biomass added in two batches separated by 3 h intervals. The pH was maintained at 4.8 with 6 M KOH during the course of hydrolysis. Chloramphenicol (Sigma-Aldrich, St. Louis, MO, USA) was added at a final concentration of 50 mg/L to minimize the risk of microbial contamination. The hydrolyzed mixture was separated by centrifugation at 8,000 rpm for 30 min, and the separated supernatant was heat-deactivated by heating the hydrolysate for 15 min at 90°C in a water bath and filtered with a 0.22-μm sterile filter (Millipore Stericup®, Millipore™, Billerica, MA, USA). The filtrate was collected and stored in the freezer until further use.

### Compositional analysis of the ACH

Glucose, xylose, arabinose, acetate, formate, and lactate in this hydrolysate mixture were analyzed using an HPLC system equipped with a Bio-Rad Aminex HPX-87H column (Bio-Rad Co., Hercules, CA, USA) as previously described [12]. The mobile phase was 5 mM H_2_SO4 at a flow rate of 0.6 mL/min, and the column temperature was maintained at 50°C. Oligosaccharides were determined by acid hydrolysis following the NREL protocol (LAP-014; www.nrel.gov/biomass/analytical_procedures.html), and the monomeric sugars produced after acid hydrolysis were quantified using HPLC (LAP-002).

Protein-derived amino acids quantification was conducted on an LC-MS system in the Department of Biochemistry and Molecular Biology at Michigan State University. The analytical methodology details have been reported elsewhere [17]. For total amino acids analysis, 100 μL of CS hydrolysate was hydrolyzed with 1 mL 6 M HCl at 110°C overnight and then dried under vacuum (SpeedVac, Eppendorf, Germany). The hydrolyzed dry sample was solubilized in 10 mL of water. Valine-d8 (1 μM) was added into the solution as an internal standard. For free amino acid analysis, the same procedure was followed with the exception of the 6 M HCl hydrolysis step.

Protein and ammonium nitrogen contents in the biomass were determined using Kjeldahl assays and a Timberline TL-1800 ammonia analyzer, respectively, at Dairy One Cooperative Inc. (Ithaca, NY). Nitrogen incorporated in the biomass during ammonolysis reactions was estimated by subtracting the total nitrogen (w/w) present in AFEX-CS from the nitrogen in UT-CS, as described previously [15].

Trace element analysis was carried out with inductively coupled plasma mass spectrometry (ICP-MS) in the ICPMS & XRF Laboratory at Michigan State University [18]. Approximately 1 mL of liquid sample was digested on a hot plate, sub-boiling in acid-cleaned Teflon Savillex beakers using 1.9 mL Optima nitric acid and 0.1 mL trace metal clean hydrofluoric acid for 24 h. After digestion, 250 μL of trace metal clean 30% hydrogen peroxide was added, and the sample was evaporated to near dryness on a hotplate. Samples were then brought up to final volume with 5 mL of 2% Optima nitric acid: visual inspection showed a complete digestion of all samples. This solution was run in the ICP-MS for full mass scan analyses. For the major element analysis, potassium (K), magnesium (Mg), calcium (Ca), phosphorus (P), and sodium (Na) samples were diluted 1:300 prior to analysis. For the trace element analysis, cobalt (Co), copper (Cu), manganese (Mn), zinc (Zn), and iron (Fe) samples were run without dilution.

Organic acids and aromatic aldehyde/ketone analyses were conducted by LC-MS/MS at Baylor University. Instrumentation and details of the applied methodology have been published elsewhere [19]. The nitrogenous compounds were identified and quantified by LC-MS/MS and GC-MS for AFEX-CS hot water extracts as reported previously by Chundawat *et al.* [15]. The composition of the ACH is presented in Table 2.

**Table 2:** **Nutrient content of AFEX-CS hydrolysate (ACH)**

**Category**	**Nutrients**	**Concentration**	**Unit**
Carbohydrates	Glucose	60	g/L
	Xylose	26	
Nitrogen	Ammonia	1.44	g/L
	Amino acids	1.44	
Vitamins*	Pantothenic acid	3.01	μM
	Pyridoxine	2.14	
	Nicotinic acid	26.78	
	Biotin	0.1	
	Thiamine	0.4	
Macro-elements	P	829.38	mg/L
	K	3886.50	
	Mg	292.86	
	Na	498.86	
	Ca	120.72	
Trace elements	Mn	3.67	mg/L
	Co	0.02	
	Cu	0.13	
	Zn	1.21	
	Fe	0.93	

*Data derived from previous study [18].

### Microorganism and seed culture

*S. cerevisiae* 424A (LNH-ST) [20], a xylose-fermenting yeast strain obtained from Purdue University, was used in this study. The seed culture was prepared by inoculating a frozen glycerol stock into 50 mL of synthetic medium (described below) using 50 g/L glucose as the sole carbon source in a 125 mL flask. The culture typically reached a cell density of 6.0 to 6.2 OD_600_ (optical density at 600 nm wavelength) after 18 h incubation at 30°C and 150 rpm. The cells were then harvested by centrifugation at 4,000 rpm for 5 min at room temperature and used as the inoculum for all reported experiments.

### Synthetic medium and fermentation

A synthetic medium (SM) with a well-defined composition was used as a seed culture and fermentation medium. The composition of the SM, as shown in Table 3, was designed to closely match the nutrient composition of AFEX-CS hydrolysate (ACH) at 6% (w/w) glucan loading (Table 2). Concentrated stock solutions of sugars, peptone (BD Bacto™ Tryptone, Franklin Lakes, NJ, USA), vitamins (Sigma-Aldrich, St. Louis, MO, USA), ammonium sulfate, and mineral salts were prepared separately and sterilized by vacuum filtration (Millipore Stericup®, 0.2 μm). The medium was adjusted to an initial pH of 5.5 with KOH and sterile filtered after the addition of all relevant components.

**Table 3:** **Nutrient content of synthetic medium mimicking AFEX-CS hydrolysate (ACH)**

**Category**	**Nutrients**	**Concentration**	**Unit**
Carbon sources	Glucose	60	g/L
	Xylose	26	
Nitrogen sources	(NH_4_)_2_SO_4_^※^	5.23	g/L
	Peptone^#^	4.35	
Vitamins	Pantothenic acid	3.01	μM
	Pyridoxine	2.14	
	Nicotinic acid	26.78	
	Biotin	0.1	
	Thiamine	0.4	
Mineral salts	KH_2_PO_4_	3319.28	mg/L
	K_2_HPO_4_	415.66	
	KCl	4341.80	
	MgCl_2_ · 6H_2_0	2449.73	
	Na_2_CO_3_	574.96	
	NaCl	634.08	
	Ca(NO_3_)_2_	711.30	
	MnCl_2_ · 4H_2_O	13.23	
	CoCl_2_ · 6H_2_O	0.06	
	CuCl_2_	0.27	
	ZnCl_2_	2.53	
	FeCl_3_	2.69	

^※^(NH_4_)_2_SO_4_ equivalent of ammonia content in the hydrolysate.

^#^Peptone equivalent to total amino acid content in the hydrolysate.

Fermentations were performed in a 25 mL Erlenmeyer flask with a working volume of 10 mL. The flasks were capped with rubber stoppers, which were pierced with a needle to vent CO_2_. Fermentations were not performed under strict anaerobic conditions; however, the air initially present in the head space of the fermentation flasks was displaced by the CO_2_ generated during fermentation. The seed was inoculated into the medium at an initial OD_600_ of 0.5, corresponding to 0.24 g/L cell mass concentration (dry weight). All fermentations were conducted in triplicate at 30°C and 150 rpm in a shaking incubator and the pH was maintained around 5.5 by periodic manual additions of 6 M KOH. As the fermentation media did not contain buffer, the pH had a tendency to decrease in the first 24 h. Therefore, we adjusted the pH to 5.5 before every sampling time. Approximately 300 μL of samples were withdrawn at designated times (0, 4, 8, 18, 24, 48, and 72 h) and frozen immediately for subsequent analysis.

Cell mass was estimated using a UV/Vis spectrophotometer (Beckman Coulter, Brea, CA) at 600-nm wavelength. One unit of absorbance is approximately equal to 0.48 g/L yeast cell biomass (dry weight). Sugars, ethanol, organic acids, glycerol, and xylitol were determined by HPLC, using the method described for compositional analysis of the hydrolysate.

The ethanol productivities for the various fermentation experiments were calculated for 24 h and/or 48 h time points, dividing the produced ethanol concentration by the respective residence time. Fermentation product yields were calculated for the first 48 h period, dividing the mass of product formed by the mass of total sugar consumed during that period. Statistical analyses for the fermentation results included standard deviations (shown in the respective tables) and a two-tailed *t*-test analysis (see Additional file 1) performed in Microsoft Excel (Microsoft, Seattle, WA).

### Effect of ACH components on fermentation

To simplify this study, all characterized AFEX pretreatment-derived biomass decomposition products were divided into five groups (Table 4): 1) nitrogenous compounds, 2) furans, 3) aliphatic acids, 4) aromatic compounds, and 5) carbohydrates.

**Table 4:** **Plant cell wall-derived decomposition products and water-soluble extractives present in AFEX-CS hydrolysate (ACH)**

**Category**	**Compound**	**Concentration (mg/L)**
Nitrogenous compounds^¶^	Feruloyl amide	1065
	*p*-Coumaroyl amide	886
	Acetamide	5674
	2-Methylpyrazine	10
	2,5-Dimethylpyrazine	1
	2,6-Dimethylpyrazine	4
	2,4-Dimethyl-1 *H*-imidazole	24
	4-Methyl-1 *H*-imidazole	95
Furan^¶^	5-Hydroxymethyl furfural	145
Aliphatic acids	Malonic acid	33
	Lactic acid	181
	*cis*-Aconitic acid	111
	Succinic acid	60
	Fumaric acid	30
	*trans*-Aconitic acid	329
	Levulinic acid	2.5
	Itaconic acid	8.2
	Acetic acid	1958
	Formic acid	517
Aromatic compounds	Vanillic acid	15
	Syringic acid	15
	Benzoic acid	59
	*p*-Coumaric acid	345
	Ferulic acid	137
	Cinnamic acid	14
	Caffeic acid	2
	Vanillin	20
	Syringaldehyde	29.5
	4-Hydroxybenzaldehyde	24
	4-Hydroxyacetophenone	3.4
Carbohydrates	Glucose	60 g/L
	Xylose	26 g/L
	Arabinose	5 g/L
	Gluco-oligomers	12 g/L
	Xylo-oligomers	18 g/L

^¶^The concentration of nitrogenous compounds and furan were calculated from the content of the analyte in dry pretreated biomass [15] based on 18% solids loading (w/v) assuming 100% solubilization into the liquid phase.

The effect of these five groups of compounds on xylose fermentation was tested individually and in combination (five groups in combination) in order to investigate their synergistic inhibitory effect. The fermentations were conducted in SM supplemented with 60 g/L glucose and 26 g/L xylose. The decomposition products in each group and their concentrations are given in Table 2, and matched their absolute abundance as found in 6% glucan loading-based ACHs. To make stock solutions of decomposition products, all compounds were dissolved in water according to the categories of aliphatic acids, aromatic acids, aromatic aldehyde/ketones, furans, imidazoles, and pyrazines at 50-fold higher concentrations and the stock solutions were sterile filtered prior to their addition into the SM. Ferulic acid, *p*-coumaric acid, amides, and carbohydrates were directly added to the fermentation media at the desired concentrations (Table 2) due to their lower solubility in water. Fermentations of SM without any decomposition products (blank) and ACHs were used as negative and positive controls, respectively. The ACH was adjusted to pH 5.5 before inoculum addition.

### Effect of nitrogenous compounds on fermentation

Fermentations were carried out in SM supplemented with 60 g/L glucose and 26 g/L xylose, respectively. The nitrogenous compounds investigated are classified into three subgroups: 1) amides, 2) pyrazines, and 3) imidazoles. All compounds in each subgroup and their concentrations tested are listed in Table 2. Fermentation of SM without any decomposition products was the control experiment (blank).

### Effect of amides and corresponding acids on fermentation

In order to compare the effect of amides and their corresponding acids on hexose/pentose sugars co-fermentation performance, feruloyl amide (6.2 mM), ferulic acid (6.2 mM), *p*-coumaroyl amide (7.5 mM), *p*-coumaric acid (7.5 mM), acetamide (28.8 mM), and acetic acid (28.8 mM) were selected for this study. The molar concentration of each compound was the total sum of the amide and its corresponding acid found in ACH at 18% solids loading (Table 2). The compound was directly dissolved in SM and the initial pH of the medium was adjusted to 5.5 before filter sterilization. The fermentation media and control experiments were as described above.

## Results and discussion

The major objective of this work is the formulation of a synthetic lignocellulosic hydrolysate (SH), as a tool to understand the effect of various components from pretreated biomass on microbial fermentation. This work provides guidelines and a methodology to formulate a detailed SH, based on the composition of industrially relevant lignocellulosic hydrolysates. The SH described in this work was designed based on the composition of ACH and was used to determine the impact of various major biomass-derived products on the performance of *S. cerevisiae* 424A (LNH-ST) fermentation. The details concerning 1) characterization of the AFEX-CS hydrolysate, 2) formulation of an SH, and 3) the impact of major hydrolysate components on yeast fermentation will be discussed here.

### Characterization of the ACH

Characterization of the ACH involves identification and quantification of 1) natively available microbial nutrients, 2) plant-derived chemicals, and 3) pretreatment-specific decomposition products. The nutrients available in the ACH are listed in Table 2 and comprise various forms of carbohydrates, nitrogenous compounds, vitamins, and minerals. The carbohydrates which could be consumed by *S. cerevisiae* 424A (LNH-ST) as a carbon source were glucose (60 g/L) and xylose (26 g/L). Other carbohydrates were found in ACH at lower abundances, including arabinose (5 g/L), glucan-derived oligomers (12 g/L), and xylan-derived oligomers (18 g/L). However, these were not categorized as nutrients, as *S. cerevisiae* 424A (LNH-ST) is not capable of using these sugars as a primary carbon source [21].

A total of 1.44 g/L of protein was estimated by LC-MS during the amino acid analysis of the ACH. Individual amino acid concentrations are shown in Additional file 1: Table S1. While the total protein concentration is fairly similar to the results of a previous study [18], the relative proportions of individual amino acids were significantly different. In this work, aspartate, valine, and proline were the most abundant amino acids, as opposed to glutamate, glycine, and alanine reported by Lau *et al.* [18]. These differences are likely related to the fact that, in this study, enzymatic hydrolysis was performed on a different source of corn stover, using different commercial enzymes. However, these changes in amino acid proportions, due to differences in feedstock and enzyme sources, did not affect the overall fermentation profiles of *S. cerevisiae* 424A (LNH-ST) grown on the hydrolysate, as the results obtained in this study are comparable to those of our previous work [10].

The free ammonium concentration found in the hydrolysate was the same as the total protein (1.44 g/L) and significantly different from the value reported previously (0.75 g/L) [18]. The concentration of free ammonium in the hydrolysate is dependent on the levels of residual ammonia left adsorbed on the biomass after AFEX pretreatment, which may vary due to differences in the relative organic acid content of the feedstock and the efficiency of ammonia removal by evaporation in the fume hood following pretreatment. However, these variations in ammonium concentration between pretreatment batches did not have significant effects on the fermentation profiles of *S. cerevisiae* 424A (LNH-ST) compared to previous studies [10,13]. This observation suggests that nitrogen is not a limiting factor for efficient fermentation of ACH. Vitamin concentrations reported in this manuscript (Table 2) were based on results previously reported by our laboratory [18]. A sensitivity analysis was carried out to evaluate the impact of vitamin concentrations on the fermentability of SH and verify if using previously reported values is a reasonable assumption for this work. For this purpose, experiments using ACH with and without 50% vitamin supplementation (based on values from Table 2) were performed. As no significant differences were observed on the fermentation profiles (data not shown) during this sensitivity analysis, it was reasonable to assume that the values obtained in the previous study could be used to estimate the vitamin composition of the SH (Table 2). It is also important to note that the goal of this study is to formulate a SH based on a typical composition of an industrially relevant biomass hydrolysate, which can vary significantly depending on the origin of the feedstock and enzymes used. Therefore, using values from our previous study was a reasonable assumption for achieving the aforementioned goals of the current study.

The mineral content of ACH was quantified by ICP-MS. Macro-elements such as P, K, and Mg were present in concentrations above the optimum range required for yeast growth defined by Jones and Greenfield (1984) [22]. These minerals are essential to all yeast and must be present in millimolar concentrations for optimal cell growth [22]. From Table 2, it is possible to observe an extremely high level of K (3886.50 mg/L), which resulted from the utilization of KOH for pH adjustment to 4.8 during enzymatic hydrolysis. Unfortunately, pH maintenance is essential to maximize enzymatic hydrolysis conversions; therefore, little could be done to avoid the accumulation of this macro-element.

Elements such as Na, Ca, and Mn are also available in concentrations above the optimum for yeast growth [22]. However, the effect of high levels of trace elements in combination with other components found in ACHs is not yet understood. Chelation effects and ionic interactions with hydrolysate components may affect the optimum range of these minerals for yeast growth [23].

Characterization of pretreatment-derived decomposition products and potential plant- derived inhibitory compounds present in the hydrolysate was performed using targeted LC- and GC-MS analysis. In our previous work, the most abundant compounds produced during AFEX pretreatment of CS were identified and quantified [15]. That work served as the platform to characterize the hydrolysate composition described here. In Table 4, these products were categorized into nitrogenous compounds, furans, aliphatic acids, aromatic compounds, and carbohydrates.

The concentration of nitrogenous compounds and furans was calculated based on the amounts present in AFEX-CS, as previously reported by Chundawat *et al.* [15]. High levels of sugars in the hydrolysate interfered with the direct quantification of these products by GC-MS, including acetamide, pyrazines, imidazoles, and furans. We considered removing those monomeric sugars from the hydrolysate prior to GC-MS analysis; however, this would have required extensive sample preparation and would thus affect the accuracy of absolute quantification of each target compound (without extensive method development, see previous study for issues encountered during typical GC-MS analysis in presence of high soluble sugar background [24]). For achieving the major goals of our current study, we have assumed that all nitrogenous compounds and furans found in AFEX-CS were totally solubilized (with 100% recovery) in the supernatant during enzymatic hydrolysis, as they are highly soluble at those concentration levels (see Table 4 for details). All other compounds presented in Table 4 were directly quantified in the hydrolysate using HPLC and LC-MS analysis.

Carbohydrates are by far the most abundant compounds in the hydrolysate, where 60 g/L glucose, 26 g/L xylose, and 5 g/L arabinose were quantified as the major carbohydrate monomers (Table 4). The concentration of carbohydrates (and other compounds) depends on the solids loading used during enzymatic hydrolysis of the pretreated biomass. In this work, 18% solids loading enzymatic hydrolysis was performed to create the ACH, giving a sufficient concentration of sugars to produce approximately 4 wt% ethanol after fermentation. This enzymatic hydrolysis condition is considered to be industrially relevant; therefore, results from this study have practical industrial relevance. However, typically under such high solids loadings, the enzymes are inhibited by high concentrations of soluble sugars [25,26], leading to the progressive accumulation of sugar oligomers derived from xylan and glucan. This likely explains the presence of 12 g/L and 18 g/L of gluco- and xylo-oligomers, respectively, in the ACH.

From Table 4, it is clear that, besides carbohydrates, the major water soluble plant-derived compounds present in ACH are feruloyl amide, *p*-coumaroyl amide, acetamide, acetic acid, *trans*-aconitic acid, formic acid, and *p*-coumaric acid. All these components are present in the hydrolysate in concentrations above 300 mg/L and, therefore, their presence at such levels can potentially impact the performance of yeast fermentation during biofuel production. The nitrogenous compounds presented in Table 4 are products of reactions between plant cell wall components and ammonia, which are produced during AFEX pretreatment [15]. For example, acetamide, feruloyl amide, and *p*-coumaroyl amide are products of ammonolysis reactions that cleave ester-bound acetates, coumarates, and ferulates, which are abundantly present in the plant cell wall of CS [27,28]. These reactions are thought to be important for the efficacy of the pretreatment, by disrupting the ester cross-links between carbohydrates and lignin, or by deacetylating the xylan backbone of hemicellulose [15,28,29]. The acid counterparts of these amides, that is, acetic acid, ferulic acid, and *p*-coumaric acid, are products of hydrolysis of the same esters, which also occur during AFEX due to the presence of hydroxyl ions in the pretreatment media [15]. Similarly to dilute acid pretreatment, formic acid is also widely produced during AFEX; however, it is formed by a different mechanism, likely via alkaline peeling reactions of polysaccharides [15,30,31]. On the other hand, *trans*-aconitic acid is not regarded as a typical AFEX pretreatment-derived decomposition product, but it is a well-known plant metabolite that is particularly abundant in grasses, including maize [15,32,33]. Therefore, its presence in a CS-derived hydrolysate at these levels is expected.

Other less abundant products present in the hydrolysate, also listed in Table 4, are included in various categories such as nitrogenous compounds, furans, aliphatic acids, and aromatic compounds. Though they are present in lower amounts in the hydrolysate, their inclusion in the SH is important because their cumulative and synergistic inhibitory effects may be significant during microbial fermentation [34].

### Formulation of a control synthetic medium

As mentioned above, ACH contains nutrients, as well as plant-derived compounds that are potentially inhibitory to microorganisms. The control synthetic medium was formulated to contain a similar level of nutrients as the biomass-derived hydrolysate, without the plant-derived inhibitory components. Table 3 summarizes the nutrient formulation of the control synthetic medium used in this work. Specifically, (NH_4_)_2_SO_4,_ peptone, and vitamins were used to match the concentrations of ammonia, protein, and vitamins, respectively, present in the hydrolysate. The concentrations of mineral elements added to the control synthetic medium were largely matched by adding a selection of salts as described in Table 3. The salts were carefully selected to avoid solubility problems during media preparation. In general, chlorine-based salts showed higher solubility in the synthetic hydrolysate than the sulfate, phosphate, or carbonate counterparts. However, a recent study by Casey *et al.* [35] revealed that chloride salts can be more detrimental to the specific xylose consumption rate of *S. cerevisiae* 424 A (LNH-ST) compared to their sulfate counterparts, for example. Therefore, high concentrations of chlorine anions in solution could negatively affect xylose fermentation. To avoid the presence of high levels of chlorine-based salts in the synthetic medium, we selected potassium salts with three different anion pairs and sodium salts with two different anion pairs (Table 3). For the same reason, we also chose to use Ca(NO_3_)_2_ instead of CaCl_2_.

**Figure 1:**
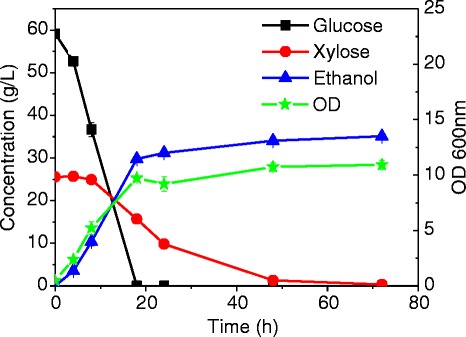
**Fermentation profile of the control synthetic medium (blank) without addition of nutrients.**

In this synthetic control medium, *S. cerevisiae* 424A completely consumed glucose and xylose in 18 and 72 h, respectively, generating ethanol at a concentration of around 35 g/L (about 80% metabolic yield) and a cell density (OD 600 nm) of approximately 12 (Figure 1). These results suggest that ACH is not limited by nitrogen, protein, or micronutrients for consuming glucose and xylose during ethanol production (though the rate of xylose uptake is significantly slower than that of glucose). However, determining the nutrient composition of AFEX pretreated biomass hydrolysates and formulating a control synthetic medium is critical to further improving microbial co-fermentations for more efficient and rapid conversion of lignocellulosic hydrolysates in the presence of inhibitory compounds. This is highlighted by the fact that xylose fermentation is affected by the nutrients level in the medium and that individual decomposition products in ACH are not very inhibitory for robust yeast species such as *S. cerevisiae* [10]. Thus, it is likely that the inhibitory effect of many of the plant-derived compounds present in the hydrolysate would not be observed in nutrient-rich media (such as Yeast Extract Peptone, YEP medium). Though the control synthetic medium formulated in this work is not exactly comparable with the actual biomass hydrolysate due to experimental limitations, the nutrient value is close enough to be considered acceptable for studying the effect of different inhibitors on strain performance. A sensitivity analysis was performed to determine how the concentration of amino acids affects cell growth and the fermentation performance. It was found that a variation of the amino acid concentration by up to two times the amount detected in the hydrolysate did not affect cell growth; however, it did improve the xylose consumption rate (data not shown).

### Inhibitory effect of different classes of compounds from ACH on *S. cerevisiae* 424A fermentation

As previously mentioned, the plant-derived compounds and pretreatment decomposition products present in ACH were divided into five groups: nitrogenous compounds, organic acids, aromatic compounds, carbohydrates, and furans (Table 4). The effect of the different groups of lignocellulose decomposition products on *S. cerevisiae* 424A fermentation was investigated and compared with the control synthetic medium (blank) formulated in this work (Table 5). The various classes of compounds identified in ACH were added to the blank medium at an abundance comparable to that in the actual hydrolysate to determine their individual and combinatorial inhibitory contribution to yeast fermentation.

**Table 5:** **Fermentation parameters for synthetic media (SM) in presence of various groups of lignocellulose decomposition products (DP)** ^**e**^

	**Biomass yield** ^**b**^ **(g/g)**	**Xylose consumption (%)**		**Ethanol productivity (g/L/h)**		**Ethanol yield** ^**c**^ **(g/g)**	**Glycerol yield (g/g)**	**Xylitol yield (g/g)**	**Acetate yield (g/g)**	**Carbon balance closure**
	**18 h**	**24 h**	**48 h**	**24 h**	**48 h**	**48 h**	**48 h**	**48 h**	**48 h**	
Blank SM^a^	0.078 ± 0.002	66.92 ± 0.18	97.08 ± 0.04	1.29 ± 0.01	0.71 ± 0.01	0.406 ± 0.001	0.058 ± 0.000	0.053 ± 0.002	0.007 ± 0.000	1.00
Blank + Nitrogenous compounds	0.067 ± 0.003	44.69 ± 0.68	86.92 ± 0.61	1.21 ± 0.00	0.69 ± 0.01	0.402 ± 0.007	0.060 ± 0.001	0.055 ± 0.003	0.007 ± 0.000	0.98
Blank + Aliphatic acids^d^	0.068 ± 0.002	58.95 ± 0.16	94.17 ± 0.04	1.23 ± 0.01	0.73 ± 0.01	0.427 ± 0.001	0.051 ± 0.000	0.042 ± 0.001	0.002 ± 0.000	1.01
Blank + Aromatic compounds	0.072 ± 0.002	68.70 ± 0.19	88.34 ± 0.04	1.29 ± 0.00	0.72 ± 0.01	0.421 ± 0.001	0.051 ± 0.000	0.032 ± 0.001	0.007 ± 0.000	1.00
Blank + Carbohydrates (oligos)	0.074 ± 0.002	54.68 ± 0.98	94.49 ± 0.50	1.28 ± 0.00	0.73 ± 0.00	0.416 ± 0.001	0.059 ± 0.000	0.043 ± 0.000	0.007 ± 0.000	1.01
Blank + Furans	0.078 ± 0.002	69.25 ± 0.19	96.90 ± 0.04	1.29 ± 0.00	0.72 ± 0.01	0.410 ± 0.001	0.059 ± 0.000	0.052 ± 0.002	0.007 ± 0.000	1.01
Blank + DP in combination^d^	0.059 ± 0.002	21.17 ± 0.09	40.05 ± 1.28	1.20 ± 0.01	0.64 ± 0.00	0.440 ± 0.000	0.045 ± 0.000	0.024 ± 0.000	−0.002 ± 0.000	0.99
Actual Hydrolysate^d^	0.065 ± 0.001	14.91 ± 0.53	43.31 ± 0.47	1.26 ± 0.01	0.73 ± 0.00	0.474 ± 0.002	0.048 ± 0.001	0.018 ± 0.000	−0.003 ± 0.001	1.06

^a^The blank was the synthetic medium without the addition of decomposition products (DP).

^b^Biomass yield was based on both glucose and xylose consumed at 18 h fermentation, when the cell density reached the maximum. One unit of absorbance at 600 nm is approximately equal to 0.48 g dry cell wt/L.

^c^Theoretical metabolic yield of ethanol for both sugars was 0.51 g EtOH/g consumed sugar.

^d^The initial concentration of acetate in the hydrolysate and synthetic medium with the addition of aliphatic acids and DP in combination was 1.9 g/L.

^e^The *t*-test results for determining statistically significant differences between the different results are presented in Additional file 1, S2, Tables S2-1 - S2-9.

From the results presented in Table 5, the nitrogenous compounds caused a significant decrease in cell biomass yield, xylose consumption rate, and 24 h ethanol productivity compared to the blank control SM (Additional file 1: Table S2-1, S2-2, and S2-4). Though this class of compounds is not usually found in most lignocellulosic hydrolysates, certain amides are produced by a variety of plants and are known to have anti-fungal effects [36]. As nitrogenous compounds are quite abundant in AFEX biomass-derived hydrolysates and limited information about their inhibitory effect on microbes is currently available in the literature, we will discuss this in more detail in the subsequent section.

Similarly to the effect of nitrogenous compounds, the xylose consumption rate and cell biomass yields were also negatively affected by the addition of aliphatic acids and aromatic compounds (Table 5). On the other hand, the ethanol metabolic yield was enhanced by the presence of these two classes of compounds, which is consistent with earlier findings in the literature [37]. As these weak acids will be present in the hydrolysate solution predominantly in their non-dissociated form, they will be permeable through the yeast cell membrane [38]. Once they enter the cytosol, the acids will dissociate and the cell will be forced to pump excess protons through the membrane to maintain homeostasis. Though low concentrations of organic acid have been observed to increase ethanol yields and fermentation rates, this benefit is lost at higher acid concentrations [39-42]. High levels of anionic acid species are also toxic to the cell and can result in cessation of growth or cell death [41,43], which does not seem to be the case for ACH. Lignin-derived aromatic compounds, such as phenols, are also known to inhibit *S. cerevisiae* growth, especially lower molecular weight phenolics. The toxicity of these compounds is dependent on the relative position (ortho, meta, or para) of the functional group in the benzene ring [44] and also on the type of functional group (for example, aldehydes, ketones, or acids). The phenolic compounds may interact with biological membranes, interfering with their function. However, the inhibition mechanism of this family of compounds is not well understood [45].

The oligomeric carbohydrates (particularly xylo-oligomers) also negatively affected xylose consumption rate in the first 24 h (18% of the control xylose consumption was reduced in the first 24 h). However, at the 48 h time point this difference was reduced to 2.7% of the control xylose consumption. As a result, the 48 h ethanol metabolic yield was only reduced by 2.5% of the control in the first 48 h. To our knowledge, xylose consumption inhibition by oligomeric carbohydrates has never been reported in the literature, and it would be interesting to determine the possible reason for this observation in a future study.

Addition of furans did not affect fermentation kinetics in great extent compared to the control (blank). The results from Table 5 show that there were no significant differences in biomass yield, 24 h ethanol productivity, and 48 h acetate yields compared to the blank SM (see Additional file 1: S2). Although the other parameters shown in Table 5 related to furan addition were statistically different from those of the blank SM, the observed difference was not very pronounced. The inhibitory effects of furans (such as furfural and hydroxymethyl furfural) on cellular metabolism have been thoroughly studied by several researchers [37,46]. These effects include oxidative damage of yeast cells by lower abundance of reducing agent concentrations (such as NADPH and NADH) and reduced activities of enzymes involved in the glycolysis pathway. From the most common furans found in lignocellulosic hydrolysates, furfural seems to be more inhibitory when compared to 5-HMF, at equivalent concentrations [47]. As AFEX pretreatment produces a low level of 5-HMF (Table 4) and practically no furfural, the concentration of this class of compounds in the hydrolysate appears to be low enough to avoid oxidative damage during yeast fermentation.

The synergistic inhibitory effect of the various classes of decomposition products (DP) was observed on xylose fermentation. The combination of all compounds (blank + DP in combination) showed a higher inhibitory effect than the aggregate value of individually added products (48 h data). This result agrees with previous reports that also observed synergies on the inhibitory effect of different compounds during yeast fermentation [47].

Among all the classes of decomposition products tested herein, nitrogenous compounds were the most inhibitory to xylose fermentation (Table 5), which could potentially be explained by their relatively higher concentration in the hydrolysate.

In the presence of aliphatic acids, about 70% decrease in acetate production was observed compared to the blank SM (Table 5). This result may be related to end-product (acetate) inhibition of the acetate synthesis pathway in yeast. Moreover, when all the decomposition products were added together, acetate was consumed by the yeast after 48 h fermentation, instead of being produced. It is possible that the yeast cells consume acetate to equilibrate the redox imbalance caused by the xylose metabolic pathway and due to the presence of high concentrations of other inhibitory compounds [48]. However, to better understand this finding, more detailed metabolomic experiments will need to be carried out in the future using SHs.

The carbon mass balance closures for the various synthetic media evaluated in Table 5 are approximately equal to 1. However, for the actual hydrolysate the carbon mass balance closes at 1.06, which means that there is 6% more carbon being formed than the carbon consumed. This observation may suggest that there are other carbon sources present in small quantities in the actual hydrolysate, which were not detected or analyzed in this study. More in-depth characterization is required to determine the minor carbon sources that contribute to this carbon mass balance closure.

### Inhibitory effect of individual families of nitrogenous compounds

**Figure 2:**
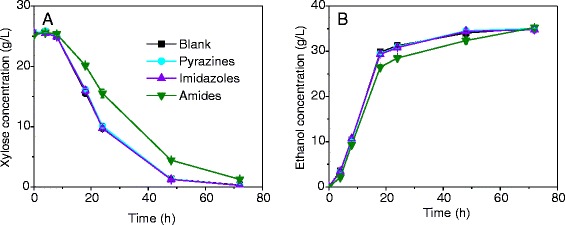
**Time course profile for xylose uptake (A) and ethanol production (B) during fermentation by** ***Saccharomyces cerevisiae*** **424A (LNH-ST) in a defined minimal synthetic medium (or blank) with addition of pyrazines, imidazoles, and amides.**

Since the effects of nitrogenous products on fermentation are particularly less well understood than the remaining categories of compounds, and because these products are specifically linked to ammonia-based pretreatment, we decided to further investigate their individual effect on the fermentation profile of *S. cerevisiae* 424A. Here, we evaluated in more detail the effect of various 1) pyrazines, 2) imidazoles, and 3) amides on xylose consumption and ethanol production rates (Table 6 and Figure 2). The results show that the addition of pyrazines or imidazoles to a well-defined SM did not significantly affect the kinetics of xylose consumption and ethanol production (*P*-value ≥ 0.05, see Additional file 1, S3, Tables S3-2 - S3-4). These two families of compounds are not present in the hydrolysate at high concentrations and therefore are likely not to have any major inhibitory effect on yeast fermentation. However, amides are present at much higher concentration in the hydrolysate, and their addition to the blank media resulted in decrease of biomass yield, xylose consumption rate, and ethanol productivity. Specifically, with the addition of amides the biomass yield, xylose consumption, and ethanol productivity were reduced from 0.068 g/g, 95%, and 0.71 g/L/h to 0.061 g/g, 80%, and 0.65 g/L/h, respectively. Though we see some level of inhibition on xylose consumption and ethanol production, the mechanisms of amide inhibition are not well understood. It is possible that phenolic amides have a similar mechanism of inhibition to the lignin-derived phenolic compounds, which tend to impact the integrity of the cell membranes when present at high concentrations [44,45].

**Table 6:** **Fermentation parameters of synthetic media (SM) with/without the addition of various nitrogenous compounds commonly found in AFEX-CS hydrolysates (ACHs)**

	**Biomass yield (g/g)**	**Xylose consumption** ^**a**^ **(%)**	**Ethanol productivity** ^**a**^ **(g/L/h)**	**Ethanol yield (g/g)**	**Glycerol yield (g/g)**	**Xylitol yield (g/g)**	**Acetate yield (g/g)**
Blank (SM)^b^	0.068 ± 0.001	95 ± 1	0.71 ± 0.01	0.411 ± 0.005	0.059 ± 0.001	0.060 ± 0.004	0.010 ± 0.001
Blank + pyrazines	0.070 ± 0.002	95 ± 0	0.72 ± 0.00	0.416 ± 0.002	0.062 ± 0.003	0.061 ± 0.001	0.010 ± 0.004
Blank + imidazoles	0.070 ± 0.002	95 ± 0	0.72 ± 0.00	0.414 ± 0.001	0.062 ± 0.003	0.060 ± 0.003	0.012 ± 0.000
Blank + amides	0.061 ± 0.000	80 ± 1	0.65 ± 0.01	0.390 ± 0.005	0.069 ± 0.001	0.056 ± 0.003	0.013 ± 0.001

^a^Both xylose consumption and ethanol volumetric productivity are shown at 48 h.

^b^Except for biomass yield, the differences between all other blank (SM) results from Tables 5 and 6 are not statistically significant (*P* > 0.05).

### Comparison between the inhibitory effects of amides and the corresponding carboxylic acids

**Figure 3:**
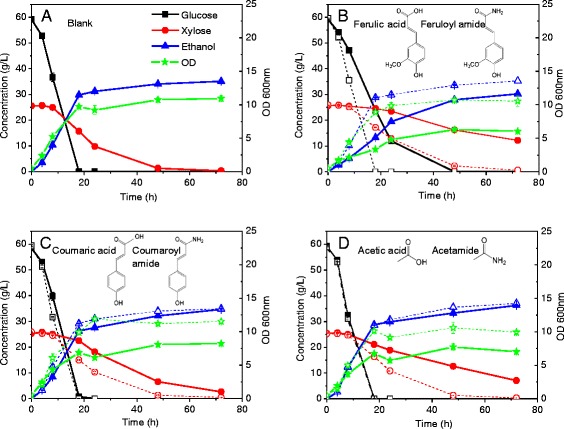
**Time course profile of co-fermentation in a minimal synthetic medium without the addition of pretreatment-based decomposition products (blank) (A) and with the addition of 6.2 mM feruloyl amide and ferulic acid (B), 7.5 mM coumaroyl amide and coumaric acid (C), and 28.8 mM acetamide and acetic acid (D) mimicking a dilute acid or ammonia pretreated lignocellulosic hydrolysate.** Solid lines depict acids; dashed lines depict amides.

Three amides (feruloyl amide, coumaroyl amide, and acetamide) present in the hydrolysate were further studied individually and their inhibition profiles were compared to their corresponding acid forms (ferulic acid, coumaric acid, and acetic acid) in the blank synthetic medium (Figure 3). Unlike the previous experiments reported herein, the concentration of amides and acids chosen for this study was not based on their actual amount in the ACH. In this case, it was assumed that all the reacting esters present in the biomass were cleaved by ammonolysis or hydrolysis reactions, respectively. As a result, the same exact molar concentrations of the acid and amide counterparts were used for each comparative inhibition experiment.

In contrast to our previous results for when the amides were added together, the individual amides did not show a substantial inhibitory effect on fermentation compared to the control. Xylose was completely consumed to undetectable levels within 72 h with maximum OD_600_ of around 12 for all the amides tested in this work (Figure 3). Therefore, the inhibitory behavior of amides is likely a synergistic effect, coupled with the fact that the total concentration of amides was higher than when present individually. The corresponding acid forms of those amide compounds, however, all showed substantial inhibition on cell growth and xylose fermentation. Among all acids, ferulic acid showed the highest inhibitory effect followed by acetic acid and coumaric acid, which was consistent with their relative abundance in the ACH (Table 4). Furthermore, ferulic acid is known to be a more potent inhibitor of yeast growth than coumaric acid, when present at similar concentrations. From the results presented in Figure 3B, the presence of ferulic acid in the fermentation media reduced the cell density by 45%. The average xylose consumption rate decreased to a very low 0.09 g/L/h (0 to 24 h), a much larger decrease than that caused by feruloyl amide (reduced to 0.55 g/L/h). Ferulic acid even affected the glucose consumption rate, which was not observed for any other decomposition product tested herein. Complete glucose consumption was only achieved after 48 h fermentation instead of 18 h, as it was in the case of the control blank medium.

From these results, it is evident that amides are less inhibitory than their corresponding acid forms, based on the same molar concentration, on yeast fermentations. Carboxylic acids permeate into the cytosol in their undissociated form when performing fermentations at pH 5.5. While in the cytosol, the acids dissociate due to the near-neutral conditions of the cytosol, decreasing the intracellular pH [38]. This effect will not be observed for amides, which typically have pKa values greater than 10. This could partially explain why AFEX pretreated biomass has greater fermentability compared to dilute acid pretreated biomass [9]. Ester hydrolysis reactions that occur during dilute acid and steam explosion pretreatments result in the formation of the organic acids studied herein, probably at similar concentrations to the ones used in this study. However, in the case of AFEX pretreatment (under the presently employed conditions) only about one third of the total available esters are hydrolyzed to yield acids, while the remaining are ammonolyzed to the less inhibitory amides. One possible way to enhance the fermentability of AFEX pretreated biomass is to further reduce the hydrolysis reaction products during pretreatment and promote conditions that improve the selectivity toward the less inhibitory ammonolysis reaction-derived products.

### Comparison between SH and ACH

**Figure 4:**
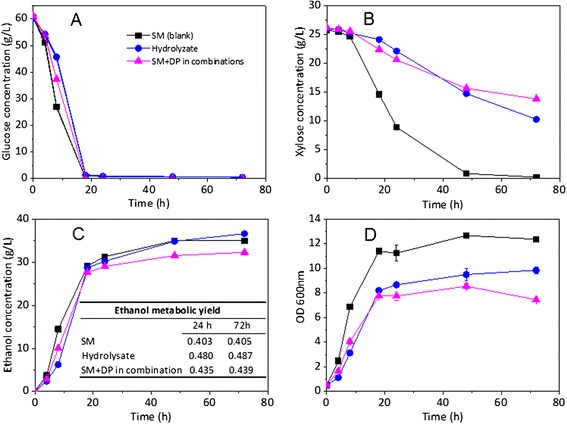
**Time course profile of co-fermentation in minimal synthetic medium (SM) with or without the addition of plant cell wall decomposition products (DP) compared to hydrolysate (ACH). (A)** and **(B)** depict glucose and xylose uptake, respectively; **(C)** depicts ethanol concentration produced; and **(D)** depicts cell density as OD 600 nm.

The fermentation profile of *S. cerevisiae* 424A in SH was compared side by side to the actual ACH as shown in Figure 4. The cell growth during fermentation in the control synthetic medium (blank) was comparable to that of the actual hydrolysate, achieving a cell density of OD_600_ 11.5 after 18 h (Figure 4D). However, cell growth in the SH, in the presence of all the decomposition products from Table 4, was greatly reduced, showing a cell density of around OD_600_ 8 after 18 h fermentation. This value represents just 68% of the cell density obtained using the blank medium.

As expected, xylose was almost completely consumed after 48 h fermentation in the control synthetic medium. The average xylose consumption rate was 0.70 g/L/h (0 to 24 h). However, the xylose consumption rates in the SH and ACH were 0.23 g/L/h and 0.28 g/L/h, respectively, which were much lower than the control rate. The lower cell density in the SH was one of the possible causes of the decreased xylose consumption rates. DP inhibition of specific xylose consumption rate and decreasing viable cell density were probably the other two reasons for the slow xylose fermentation [13]. Regarding the ethanol yield, SH and ACH results were statistically different (0.439 g/g and 0.486 g/g, respectively) and this difference represents about 10% of the ACH ethanol yield. In both these cases, the ethanol yields were significantly higher than the control (0.405 g/g). This increased ethanol metabolic yield in the presence of AFEX pretreatment-derived decomposition products is consistent with our previous observations and other reports [10,13,49]. The final ethanol concentrations achieved in the ACH, control synthetic medium, and SH were 38 g/L, 35 g/L, and 32 g/L, respectively. During the first 18 h period, glucose and xylose consumption were equivalent for both media, and only after 18 h fermentation was it possible to observe significant differences in xylose consumption and, consequently, in ethanol production. Therefore, the higher ethanol yields observed for the actual biomass hydrolysate seem to be related to better xylose fermentation.

For an ideal SH, one would expect identical cell growth behavior, sugar consumption rates and ethanol yields to those observed for the actual hydrolysate. The differences in cell growth profile between the SH and the ACH may be due to incomplete evaluation of the composition of the actual hydrolysate, which is very complex and presents various analytical challenges. Possible improvements for future versions of the SH may include the analysis of redox co-factors present in plant biomass (for example, NAD(P)H), which could potentially help the yeast cells to improve their fermentation performance. Also, the higher concentration of chlorine-containing salts in the SH might be another possible factor that could have caused such a negative impact (Tables 2 and 3). Therefore, optimizing the choice of salts to closely match the mineral content of the ACH would help improve the performance of the SH. Nevertheless, the SH presented in this study was successfully used to evaluate the relative levels of inhibition associated with the various classes of compounds that are present in the actual ACH. Moreover, as we performed a detailed characterization of the amino acids present in the ACH (Additional file 1: Table S1), it is possible to formulate a well-defined synthetic medium by the addition of individual amino acids, at the respective concentrations, in contrast to peptone. The utilization of defined synthetic media will be important for future *multi-omics* studies that will help us understand the mechanisms of inhibition under well-controlled experimental conditions.

## Conclusion

In this work, nutrients and decomposition products present in ACH were characterized with the goal of formulating a synthetic hydrolysate, which will be used in *multi-omics* analysis for understanding the inhibition mechanisms of the lignocellulosic hydrolysate. This work also provides an example showing how synthetic lignocellulosic hydrolysates derived from other pretreatment technologies (such as dilute acid and steam explosion) can be formulated.

The ACH contained high levels of nitrogenous compounds, notably phenolic amides and acetamide. Due to their presence at high concentrations, their observed inhibitory effect on xylose consumption and ethanol production was the most significant among all the families of compounds tested herein, which included aliphatic acids, furans, lignin-derived phenolic compounds, and oligomeric carbohydrates. However, when comparing the inhibition due to amides at the same molar concentrations as their acid counterparts, we observed that amides are significantly less inhibitory to both glucose and xylose fermentation than the acids. The reduced inhibitory effect of amides is a major advantage of AFEX- and ammonia-based pretreatments over other pretreatment technologies that mainly produce carboxylic acids as decomposition products. Because of the reduced production of carboxylic acids and furans, notably furfural and 5-HMF, the ACH is easily fermentable without any detoxification.

Although we were able to identify the major groups of inhibitory compounds present in the ACH, the SH did not exactly match the performance of the actual hydrolysate. The cell density in SH was considerably lower than in the actual hydrolysate and, as a consequence, the xylose consumption rate was also slightly reduced. However, the proposed SH was instrumental in identifying the inhibitory effect of various classes of compounds present in the hydrolysate and their relative contribution to the overall inhibition. Due to the complexity of the lignocellulosic hydrolysate composition, we will likely develop more representative versions of the SH as we learn more about the composition of actual hydrolysates. The SH formulation will be instrumental in future *multi-omics* studies to understand the nature of AFEX pretreatment-specific decomposition products and how they inhibit yeast and bacteria, so that we can engineer better strains to maximize biofuel yields and productivity.

### Endnote

^a^TM - AFEX is a trademark of MBI International, Lansing, Michigan.

## Additional file

Additional file 1:
**S1.** Amino acid content of AFEX-CS hydrolysate (ACH) and Peptone. **Table S1.** Analysis of amino acid content in AFEX-CS hydrolysate (ACH) and peptone. Calculation of the peptone equivalent concentration to meet the total amino acid value present in ACH. **S2.** Statistical analysis for Table 5. **Table S2-1.** Map of *P*-value range from *t*-test for biomass yield results. **Table S2-2.** Map of *P*-value range from *t*-test for 24 h xylose consumption results. **Table S2-3.** Map of *P*-value range from *t*-test for 48 h xylose consumption results. **Table S2-4.** Map of *P*-value range from *t*-test for 24 h ethanol productivity results. **Table S2-5.** Map of *P*-value range from *t*-test for 48 h ethanol productivity results.**S3.** Statistical analysis for Table 6. **Table S3-1.** Map of *P*-value from *t*-test for 18 h biomass yield results. **Table S3-2.** Map of *P*-value from *t*-test for 48 h xylose consumption results. **Table S3-3.** Map of *P*-value from *t*-test for 48 h ethanol productivity results. **Table S3-4.** Map of *P*-value from *t*-test for 48 h ethanol yield results. **Table S3-5.** Map of *P*-value from *t*-test for 48 h glycerol yield results. **Table S3-6.** Map of *P*-value from *t*-test for 48 h xylitol yield results. **Table S3-7.** Map of *P*-value from *t*-test for 48 h acetate yield results.

## Abbreviations

AA: amino acid
ACH: AFEX™ pretreated corn stover hydrolysate
AFEX: Ammonia Fiber Expansion
CS: corn stover
DP: decomposition products
5-HMF: 5-hydroxymethylfurfural
SH: synthetic lignocellulosic hydrolysate
SM: synthetic medium
UT-CS: untreated corn stover
YEP: Yeast extract peptone

## References

[1] 109^th^ US Congress. Energy and Policy Act of 2005*,* PUBLIC LAW 109-58-AUG 8, 2005, 119 STAT:594-1143

[2] Yang B, Wyman CE (2008). Pretreatment: the key to unlocking low-cost cellulosic ethanol. Biofuels Bioprod Biorefin.

[3] Rubin EM (2008). Genomics of cellulosic biofuels. Nature.

[4] da Costa SL, Chundawat SP, Balan V, Dale BE (2009). 'Cradle-to-grave' assessment of existing lignocellulose pretreatment technologies. Curr Opin Biotechnol.

[5] Palmqvist E, Hahn-Hagerdal B (2000). Fermentation of lignocellulosic hydrolysates. II: inhibitors and mechanisms of inhibition. Bioresour Technol.

[6] Oliva JM, Saez F, Ballesteros I, Gonzalez A, Negro MJ, Manzanares P (2003). Effect of lignocellulosic degradation compounds from steam explosion pretreatment on ethanol fermentation by thermotolerant yeast *Kluyveromyces marxianus*. Appl Biochem Biotechnol.

[7] Klinke HB, Olsson L, Thomsen AB, Ahring BK (2003). Potential inhibitors from wet oxidation of wheat straw and their effect on ethanol production of *Saccharomyces cerevisiae*: Wet oxidation and fermentation by yeast. Biotechnol Bioeng.

[8] Mielenz JR (2009). Biofuels: Methods and Protocols.

[9] Lau M, Gunawan C, Dale B (2009). The impacts of pretreatment on the fermentability of pretreated lignocellulosic biomass: a comparative evaluation between ammonia fiber expansion and dilute acid pretreatment. Biotechnol Biofuels.

[10] Lau MW, Dale BE (2009). Cellulosic ethanol production from AFEX-treated corn stover using *Saccharomyces cerevisiae* 424A(LNH-ST). Proc Natl Acad Sci.

[11] Lau MW, Dale BE, Balan V (2008). Ethanolic fermentation of hydrolysates from ammonia fiber expansion (AFEX) treated corn stover and distillers grain without detoxification and external nutrient supplementation. Biotechnol Bioeng.

[12] Lau MW, Dale BE (2010). Effect of primary degradation-reaction products from Ammonia Fiber Expansion (AFEX)-treated corn stover on the growth and fermentation of *Escherichia coli* KO11. Bioresour Technol.

[13] Jin M, Balan V, Gunawan C, Dale BE (2012). Quantitatively understanding reduced xylose fermentation performance in AFEXTM treated corn stover hydrolysate using *Saccharomyces cerevisiae* 424A (LNH-ST) and *Escherichia coli* KO11. Bioresour Technol.

[14] Balan V, Bals B, Chundawat SP, Marshall D, Dale BE. Lignocellulosic biomass pretreatment using AFEX. In Biofuels: Methods and Protocols. Volume 581; 2009: 61–77: Methods in Molecular Biology. 10.1007/978-1-60761-214-8_5 19768616

[15] Chundawat SPS, Vismeh R, Sharma LN, Humpula JF, da Costa SL, Chambliss CK (2010). Multifaceted characterization of cell wall decomposition products formed during ammonia fiber expansion (AFEX) and dilute acid based pretreatments. Bioresour Technol.

[16] Chundawat SPS, Lipton MS, Purvine SO, Uppugundla N, Gao D, Balan V (2011). Proteomics-based compositional analysis of complex cellulase-hemicellulase mixtures. J Proteome Res.

[17] Gu L, Jones AD, Last RL (2007). LC-MS/MS assay for protein amino acids and metabolically related compounds for large-scale screening of metabolic phenotypes. Anal Chem.

[18] Lau MW, Bals BD, Chundawat SPS, Jin M, Gunawan C, Balan V (2012). An integrated paradigm for cellulosic biorefineries: utilization of lignocellulosic biomass as self-sufficient feedstocks for fuel, food precursors and saccharolytic enzyme production. Energ Environ Sci.

[19] Sharma LN, Becker C, Chambliss CK. Analytical characterization of fermentation inhibitors in biomass pretreatment samples using liquid chromatography, UV-visible spectroscopy, and tandem mass spectrometry. In Biofuels: Methods and Protocols. Volume 581. Edited by Mielenz JR; 2009: 125–143: Methods in Molecular Biology 10.1007/978-1-60761-214-8_10 19768621

[20] Ho NWY, Chen ZD, Brainard AP (1998). Genetically engineered *Sacccharomyces* yeast capable of effective cofermentation of glucose and xylose. Appl Environ Microbiol.

[21] Sedlak M, Ho N (2004). Production of ethanol from cellulosic biomass hydrolysates using genetically engineered *Saccharomyces* yeast capable of cofermenting glucose and xylose. Appl Biochem Biotechnol.

[22] Jones RP, Greenfield PF (1984). A review of yeast ionic nutrition. Part 1: Growth and fermentation requirements. Process Biochem.

[23] Chandrasena G, Walker GM, Staines HJ (1997). Use of response surfaces to investigate metal ion interactions in yeast fermentations. J Am Soc Brew Chem.

[24] Humpula JF, Chundawat SPS, Vismeh R, Jones AD, Balan V, Dale BE (2011). Rapid quantification of major reaction products formed during thermochemical pretreatment of lignocellulosic biomass using GC-MS. J Chromatogr B.

[25] Teugjas H, Valjamae P (2013). Product inhibition of cellulases studied with 14C-labeled cellulose substrates. Biotechnol Biofuels.

[26] Baumann M, Borch K, Westh P (2011). Xylan oligosaccharides and cellobiohydrolase I (TrCel7A) interaction and effect on activity. Biotechnol Biofuels.

[27] Bunzel M, Ralph J, Steinhart H (2004). Phenolic compounds as cross-links of plant derived polysaccharides. Czech J Food Sci.

[28] Mitchell DJ, Grohmann K, Himmel ME, Dale BE, Schroeder HA (1990). Effect of the degree of acetylation on the enzymatic digestion of acetylated xylans. J Wood Chem Technol.

[29] Chundawat SPS, Donohoe BS, da Costa SL, Elder T, Agarwal UP, Lu F (2011). Multi-scale visualization and characterization of lignocellulosic plant cell wall deconstruction during thermochemical pretreatment. Energ Environ Sci.

[30] Simmons B (2011). Chemical and Biochemical Catalysis for Next Generation Biofuels.

[31] Himmel ME, Ding S-Y, Johnson DK, Adney WS, Nimlos MR, Brady JW (2007). Biomass recalcitrance: engineering plants and enzymes for biofuels production. Science.

[32] Burau R, Stout P (1965). Trans-aconitic acid in range grasses in early spring. Science.

[33] Brauer D, Teel MR (1982). Metabolism of trans-aconitic acid in maize. Plant Physiol.

[34] Moon NJ (1983). Inhibition of the growth of acid tolerant yeasts by acetate, lactate and propionate and their synergistic mixtures. J Appl Bacteriol.

[35] Casey E, Mosier N, Adamec J, Stockdale Z, Ho N, Sedlak M (2013). Effect of salts on the Co-fermentation of glucose and xylose by a genetically engineered strain of *Saccharomyces cerevisiae*. Biotechnol Biofuels.

[36] Lee D, Park Y, Kim M-R, Jung H, Seu Y, Hahm K-S (2004). Anti-fungal effects of phenolic amides isolated from the root bark of Lycium chinense. Biotechnol Lett.

[37] Almeida JRM, Modig T, Petersson A, Hähn-Hägerdal B, Lidén G, Gorwa-Grauslund MF (2007). Increased tolerance and conversion of inhibitors in lignocellulosic hydrolysates by *Saccharomyces cerevisiae*. J Chem Technol Biotechnol.

[38] Bellissimi E, Van Dijken JP, Pronk JT, Van Maris AJA (2009). Effects of acetic acid on the kinetics of xylose fermentation by an engineered, xylose-isomerase-based *Saccharomyces cerevisiae* strain. FEMS Yeast Res.

[39] Pampulha ME, Loureiro-Dias MC (2000). Energetics of the effect of acetic acid on growth of *Saccharomyces cerevisiae*. FEMS Microbiol Lett.

[40] Abbott DA, Ingledew WM (2004). Buffering capacity of whole corn mash alters concentrations of organic acids required to inhibit growth of *Saccharomyces cerevisiae* and ethanol production. Biotechnol Lett.

[41] Graves T, Narendranath N, Dawson K, Power R (2006). Effect of pH and lactic or acetic acid on ethanol productivity by *Saccharomyces cerevisiae* in corn mash. J Ind Microbiol Biot.

[42] Keating JD, Panganiban C, Mansfield SD (2006). Tolerance and adaptation of ethanologenic yeasts to lignocellulosic inhibitory compounds. Biotechnol Bioeng.

[43] Pampulha ME, Loureiro-Dias MC (1989). Combined effect of acetic acid, pH and ethanol on intracellular pH of fermenting yeast. Appl Microbiol Biotechnol.

[44] Larsson S, Quintana-Sáinz A, Reimann A, Nilvebrant N-O, Jönsson L (2000). Influence of lignocellulose-derived aromatic compounds on oxygen-limited growth and ethanolic fermentation by *Saccharomyces cerevisiae*. Appl Biochem Biotechnol.

[45] Heipieper HJ, Weber FJ, Sikkema J, Keweloh H, de Bont JAM (1994). Mechanisms of resistance of whole cells to toxic organic solvents. Trends Biotechnol.

[46] Gorsich SW, Dien BS, Nichols NN, Slininger PJ, Liu ZL, Skory CD (2006). Tolerance to furfural-induced stress is associated with pentose phosphate pathway genes *ZWF1*, *GND1*, *RPE1*, and *TKL1* in *Saccharomyces cerevisiae*. Appl Microbiol Biotechnol.

[47] Iwaki A, Kawai T, Yamamoto Y, Izawa S (2013). Biomass conversion inhibitors furfural and 5-hydroxymethylfurfural induce formation of messenger RNP granules and attenuate translation activity in *Saccharomyces cerevisiae*. Appl Environ Microbiol.

[48] Wei N, Quarterman J, Kim SR, Cate JHD, Jin Y-S (2013). Enhanced biofuel production through coupled acetic acid and xylose consumption by engineered yeast. Nat Commun.

[49] Jin M, Lau MW, Balan V, Dale BE (2010). Two-step SSCF to convert AFEX-treated switchgrass to ethanol using commercial enzymes and *Saccharomyces cerevisiae* 424A(LNH-ST). Bioresour Technol.

