# Supplementary material for: Designer synthetic media for studying microbial-catalyzed biofuel production
Source: Biotechnol Biofuels. 2015 Jan 22;8:1. doi: 10.1186/s13068-014-0179-6 (PMC4311453; doi:10.1186/s13068-014-0179-6)
Supplement: Additional file 1: — S1. Amino acid content of AFEX-CS hydrolysate (ACH) and Peptone. Table S1. Analysis of amino acid content in AFEX-CS hydrolysate (ACH) and peptone. Calculation of the peptone equivalent concentration to meet the total amino acid value present in ACH. S2. Statistical analysis for Table 5. Table S2-1. Map of P-value range from t-test for biomass yield results. Table S2-2. Map of P-value range from t-test for 24 h xylose consumption results. Table S2-3. Map of P-value range from t-test for 48 h xylose consumption results. Table S2-4. Map of P-value range from t-test for 24 h ethanol productivity results. Table S2-5. Map of P-value range from t-test for 48 h ethanol productivity results.S3. Statistical analysis for Table 6. Table S3-1. Map of P-value from t-test for 18 h biomass yield results. Table S3-2. Map of P-value from t-test for 48 h xylose consumption results. Table S3-3. Map of P-value from t-test for 48 h ethanol productivity results. Table S3-4. Map of P-value from t-test for 48 h ethanol yield results. Table S3-5. Map of P-value from t-test for 48 h glycerol yield results. Table S3-6. Map of P-value from t-test for 48 h xylitol yield results. Table S3-7. Map of P-value from t-test for 48 h acetate yield results. [file 13068_2014_179_MOESM1_ESM.docx]

**Additional file 1**

**S1 – Amino acid content of AFEX-CS hydrolysate (ACH) and Peptone**

**Table S1 –** Analysis of amino acid content in AFEX-CS hydrolysate (ACH) and peptone. Calculation of the peptone equivalent concentration to meet the total amino acid value present in ACH.

|  | **ACH** | | **Peptone** |
| --- | --- | --- | --- |
| **Amino acid**  **(AA)** | **Concentration (mg/L)** | **% of total amino acid** | **% of total amino acid*** |
| Ala | 72.94 | 5.07% | 3.2% |
| Arg | 23.78 | 1.65% | 2.7% |
| Asn | 7.76 | 0.54% | N/A^¥^ |
| Asp | 196.06 | 13.62% | 5.2% |
| Cys | 0 | 0.00% | N/A |
| Gln | 3.99 | 0.28% | N/A |
| Glu | 141.97 | 9.86% | 15.1% |
| Gly | 86.15 | 5.98% | 1.7% |
| His | 30.73 | 2.13% | 1.9% |
| Ile | 43.27 | 3.01% | 5.5% |
| Leu | 98.95 | 6.87% | 7.5% |
| Lys | 74.89 | 5.20% | 6.2% |
| Met | 34.97 | 2.43% | 2.1% |
| Phe | 57.82 | 4.02% | 5.2% |
| Pro | 151.90 | 10.55% | 6.6% |
| Ser | 83.25 | 5.78% | 2.2% |
| Trp | 3.89 | 0.27% | N/A |
| Tyr | 58.68 | 4.08% | 1.3% |
| Val | 165.25 | 11.48% | 5.9% |
| Hpro | 0 | 0.00% | N/A |
| Thr | 103.24 | 7.17% | 1.8% |
|  |  |  |  |
| **Total AA in hydrolysate** | 1439.49 | mg/L |  |
| **Peptone equivalent^#^** | 4347.17 | mg/L |  |

^*^ Provided by Becton, Dickinson and Company

^¥^ N/A: Not Available

^#^ Peptone equivalent (mg/L) = total AA (in hydrolysate)/(Amino-N in peptone *6.25). The Amino-N composition of peptone is 5.3 wt%, as provided by Becton, Dickinson and Company.

S2 – Statistical analysis for Table 5

Legend for t-test tables:

| 1. Blank SM | |  |
| --- | --- | --- |
| 1. Blank+Nitrogenous compounds | |  |
| 1. Blank+Aliphatic acids | |  |
| 1. Blank+Aromatic compounds | |  |
| 1. Blank+Carbohydrates (oligos) | |  |
| 1. Blank+furans | |  |
| 1. Blank+DP in combination | |  |
| 1. Actual Hydrolysate | |  |
| *P* < 0.05 | Differences are statistically significant | |
| *P* ≥ 0.05 | Differences are not statistically significant | |

Table S2-1 Map of *p*-value range from t-test for biomass yield results

|  | A | B | C | D | E | F | G | H |
| --- | --- | --- | --- | --- | --- | --- | --- | --- |
| A |  |  |  |  |  |  |  |  |
| B |  |  |  |  |  |  |  |  |
| C |  |  |  |  |  |  |  |  |
| D |  |  |  |  |  |  |  |  |
| E |  |  |  |  |  |  |  |  |
| F |  |  |  |  |  |  |  |  |
| G |  |  |  |  |  |  |  |  |
| H |  |  |  |  |  |  |  |  |

Table S2-2 Map of *p*-value range from t-test for 24h xylose consumption results

|  | A | B | C | D | E | F | G | H |
| --- | --- | --- | --- | --- | --- | --- | --- | --- |
| A |  |  |  |  |  |  |  |  |
| B |  |  |  |  |  |  |  |  |
| C |  |  |  |  |  |  |  |  |
| D |  |  |  |  |  |  |  |  |
| E |  |  |  |  |  |  |  |  |
| F |  |  |  |  |  |  |  |  |
| G |  |  |  |  |  |  |  |  |
| H |  |  |  |  |  |  |  |  |

Table S2-3 Map of *p*-value range from t-test for 48h xylose consumption results

|  | A | B | C | D | E | F | G | H |
| --- | --- | --- | --- | --- | --- | --- | --- | --- |
| A |  |  |  |  |  |  |  |  |
| B |  |  |  |  |  |  |  |  |
| C |  |  |  |  |  |  |  |  |
| D |  |  |  |  |  |  |  |  |
| E |  |  |  |  |  |  |  |  |
| F |  |  |  |  |  |  |  |  |
| G |  |  |  |  |  |  |  |  |
| H |  |  |  |  |  |  |  |  |

Table S2-4 Map of *p*-value range from t-test for 24h ethanol productivity results

|  | A | B | C | D | E | F | G | H |
| --- | --- | --- | --- | --- | --- | --- | --- | --- |
| A |  |  |  |  |  |  |  |  |
| B |  |  |  |  |  |  |  |  |
| C |  |  |  |  |  |  |  |  |
| D |  |  |  |  |  |  |  |  |
| E |  |  |  |  |  |  |  |  |
| F |  |  |  |  |  |  |  |  |
| G |  |  |  |  |  |  |  |  |
| H |  |  |  |  |  |  |  |  |

Table S2-5 Map of *p*-value range from t-test for 48h ethanol productivity results

|  | A | B | C | D | E | F | G | H |
| --- | --- | --- | --- | --- | --- | --- | --- | --- |
| A |  |  |  |  |  |  |  |  |
| B |  |  |  |  |  |  |  |  |
| C |  |  |  |  |  |  |  |  |
| D |  |  |  |  |  |  |  |  |
| E |  |  |  |  |  |  |  |  |
| F |  |  |  |  |  |  |  |  |
| G |  |  |  |  |  |  |  |  |
| H |  |  |  |  |  |  |  |  |

Table S2-6 Map of *p*-value range from t-test for 48h ethanol yield results

|  | A | B | C | D | E | F | G | H |
| --- | --- | --- | --- | --- | --- | --- | --- | --- |
| A |  |  |  |  |  |  |  |  |
| B |  |  |  |  |  |  |  |  |
| C |  |  |  |  |  |  |  |  |
| D |  |  |  |  |  |  |  |  |
| E |  |  |  |  |  |  |  |  |
| F |  |  |  |  |  |  |  |  |
| G |  |  |  |  |  |  |  |  |
| H |  |  |  |  |  |  |  |  |

Table S2-7 Map of *p*-value range from t-test for 48h glycerol yields results

|  | A | B | C | D | E | F | G | H |
| --- | --- | --- | --- | --- | --- | --- | --- | --- |
| A |  |  |  |  |  |  |  |  |
| B |  |  |  |  |  |  |  |  |
| C |  |  |  |  |  |  |  |  |
| D |  |  |  |  |  |  |  |  |
| E |  |  |  |  |  |  |  |  |
| F |  |  |  |  |  |  |  |  |
| G |  |  |  |  |  |  |  |  |
| H |  |  |  |  |  |  |  |  |

Table S2-8 Map of *p*-value range from t-test for 48h xylitol yields results

|  | A | B | C | D | E | F | G | H |
| --- | --- | --- | --- | --- | --- | --- | --- | --- |
| A |  |  |  |  |  |  |  |  |
| B |  |  |  |  |  |  |  |  |
| C |  |  |  |  |  |  |  |  |
| D |  |  |  |  |  |  |  |  |
| E |  |  |  |  |  |  |  |  |
| F |  |  |  |  |  |  |  |  |
| G |  |  |  |  |  |  |  |  |
| H |  |  |  |  |  |  |  |  |

Table S2-9 Map of *p*-value range from t-test for 48h acetate yields results

|  | A | B | C | D | E | F | G | H |
| --- | --- | --- | --- | --- | --- | --- | --- | --- |
| A |  |  |  |  |  |  |  |  |
| B |  |  |  |  |  |  |  |  |
| C |  |  |  |  |  |  |  |  |
| D |  |  |  |  |  |  |  |  |
| E |  |  |  |  |  |  |  |  |
| F |  |  |  |  |  |  |  |  |
| G |  |  |  |  |  |  |  |  |
| H |  |  |  |  |  |  |  |  |

S3 – Statistical analysis for Table 6

Legend for t-test tables:

| A - Blank (SM) | | | |
| --- | --- | --- | --- |
| B - Blank+Pyrazines | | | |
| C - Blank+Imidazoles | | | |
| D - Blank+Amides | | | |
| *P* < 0.05 | Differences are statistically significant |  |  |
| *P* ≥ 0.05 | Differences are not statistically significant |  |  |

Table S3-1 Map of *p*-value range from t-test for 18h biomass yield results.

|  | A | B | C | D |
| --- | --- | --- | --- | --- |
| A |  |  |  |  |
| B |  |  |  |  |
| C |  |  |  |  |
| D |  |  |  |  |

Table S3-2 Map of *p*-value range from t-test for 48h xylose consumption results.

|  | A | B | C | D |
| --- | --- | --- | --- | --- |
| A |  |  |  |  |
| B |  |  |  |  |
| C |  |  |  |  |
| D |  |  |  |  |

Table S3-3 Map of *p*-value range from t-test for 48h ethanol productivity results.

|  | A | B | C | D |
| --- | --- | --- | --- | --- |
| A |  |  |  |  |
| B |  |  |  |  |
| C |  |  |  |  |
| D |  |  |  |  |

Table S3-4 Map of *p*-value range from t-test for 48h ethanol yield results.

|  | A | B | C | D |
| --- | --- | --- | --- | --- |
| A |  |  |  |  |
| B |  |  |  |  |
| C |  |  |  |  |
| D |  |  |  |  |

Table 3-5 Map of *p*-value range from t-test for 48h glycerol yield results.

|  | A | B | C | D |
| --- | --- | --- | --- | --- |
| A |  |  |  |  |
| B |  |  |  |  |
| C |  |  |  |  |
| D |  |  |  |  |

Table S3-6 Map of *p*-value range from t-test for 48h xylitol yield results.

|  | A | B | C | D |
| --- | --- | --- | --- | --- |
| A |  |  |  |  |
| B |  |  |  |  |
| C |  |  |  |  |
| D |  |  |  |  |

Table S3-7 Map of *p*-value range from t-test for 48h acetate yield results.

|  | A | B | C | D |
| --- | --- | --- | --- | --- |
| A |  |  |  |  |
| B |  |  |  |  |
| C |  |  |  |  |
| D |  |  |  |  |
